# Deploying a national clinical text processing infrastructure

**DOI:** 10.1093/jamia/ocad249

**Published:** 2023-12-26

**Authors:** Kimberly F McManus, Johnathon Michael Stringer, Neal Corson, Samah Fodeh, Steven Steinhardt, Forrest L Levin, Asqar S Shotqara, Joseph D’Auria, Elliot M Fielstein, Glenn T Gobbel, John Scott, Jodie A Trafton, Tamar H Taddei, Joseph Erdos, Suzanne R Tamang

**Affiliations:** Department of Veterans Affairs, Office of the CTO, Washington, DC 20571, United States; Division of Immunology and Rheumatology, Department of Medicine, Stanford University, Stanford, CA 94304, United States; Department of Veterans Affairs, San Diego, CA 92108, United States; Department of Veterans Affairs, West Haven, CT 06516, United States; Yale School of Medicine, New Haven, CT 06510, United States; Evergreen Design LLC, Guilford, CT 06437, United States; Evergreen Design LLC, Guilford, CT 06437, United States; Department of Veterans Affairs, Center for Innovation to Implementation (Ci2i), Palo Alto, CA 94304, United States; Product Engineering, Department of Veterans Affairs, Austin, TX 78741, United States; Department of Veterans Affairs, Office of Mental Health and Suicide Prevention, Veterans Health Administration, Nashville, TN 37212, United States; Department of Biomedical Informatics, Vanderbilt University Medical Center, Nashville, TN 37203, United States; Department of Biomedical Informatics, Vanderbilt University Medical Center, Nashville, TN 37203, United States; Department of Veterans Affairs, Clinical Informatics and Data Management Office, Veterans Health Administration, Washington, DC 20571, United States; Department of Veterans Affairs, Office of Mental Health and Suicide Prevention, Program Evaluation Resource Center, Palo Alto, CA 94304, United States; Department of Veterans Affairs, West Haven, CT 06516, United States; Yale School of Medicine, New Haven, CT 06510, United States; Department of Veterans Affairs, West Haven, CT 06516, United States; Yale School of Medicine, New Haven, CT 06510, United States; Division of Immunology and Rheumatology, Department of Medicine, Stanford University, Stanford, CA 94304, United States; Department of Veterans Affairs, Office of Mental Health and Suicide Prevention, Program Evaluation Resource Center, Palo Alto, CA 94304, United States

**Keywords:** natural language processing, machine learning, mental health, delivery of health care

## Abstract

**Objectives:**

Clinical text processing offers a promising avenue for improving multiple aspects of healthcare, though operational deployment remains a substantial challenge. This case report details the implementation of a national clinical text processing infrastructure within the Department of Veterans Affairs (VA).

**Methods:**

Two foundational use cases, cancer case management and suicide and overdose prevention, illustrate how text processing can be practically implemented at scale for diverse clinical applications using shared services.

**Results:**

Insights from these use cases underline both commonalities and differences, providing a replicable model for future text processing applications.

**Conclusions:**

This project enables more efficient initiation, testing, and future deployment of text processing models, streamlining the integration of these use cases into healthcare operations. This project implementation is in a large integrated health delivery system in the United States, but we expect the lessons learned to be relevant to any health system, including smaller local and regional health systems in the United States.

## Introduction

Clinical text processing has the potential to improve the delivery, quality, and safety of healthcare. There are many research applications, including the extraction of clinical concepts and other information from clinical progress notes that may be under coded or absent from the structured data^1–3^; however, examples of health systems operationalizing these tools for real-world clinical decision-making, population health management, and quality measurement are limited.^4^^,^^5^ This represents a missed opportunity to turn rich, unstructured data into actionable information that can be used for quality and performance initiatives within a health system.^4–6^

The Department of Veterans Affairs (VA) has a long history of electronic health record (EHR) innovation. The VA’s on-prem Corporate Data Warehouse (CDW) has registered over 24 million patients and contains EHR data that dates to the year 2000. The next step in VA’s data journey is the Health Data and Analytics Platform (HDAP), a scalable, cloud-native enterprise data analytics platform with the goal of enabling big data projects and lowering the barrier to developing, deploying, and operationalizing projects, such as text processing models, for clinical decision support.

Our primary project objective is to leverage this new platform for development of text processing models and, crucially, simplify the transition from prototyping and research to deploying models at scale and integrating into clinical workflows. A secondary objective is to reduce silos and project duplication by facilitating the discovery and utilization of existing text processing models and their structured text outputs across different program offices. Here, we present 2 initial use cases focused on some of VA’s top clinical priorities: cancer care, suicide, and overdose prevention.

## Methods

### Case study: improving care and safety in the VHA using the coordinated care tracking system

The Coordinated Care Tracking System (CCTS) ensures timely detection, triage, and longitudinal follow-up of cancer.^7^ It is a web-based, electronic medical record (EMR)-linked tracking system for care coordination and management of patients with existing or suspected complex disease states, including lung cancer. The CCTS was migrated to HDAP to allow broader implementation and enhanced clinical decision support. It is available nationally for all 130 VA VistA EHR installations and is currently adopted at 60 VA Medical Centers. The goal of CCTS text processing is to identify lung nodules from free text in radiology reports. Reports identified as including potentially relevant lung nodules, which were not already flagged for follow-up, would then be surfaced within the CCTS application for human review of whether additional follow-up is necessary.

### Case study: improving overdose and suicide prediction with clinical text processing

Despite well-established evidence for the role of social determinants of health (SDoH) as risk factors for suicide and overdose, state-of-the-art algorithms draw almost exclusively from clinical risk factors. To address this gap in clinical risk assessment, the HDAP pipeline was designed to extract SDoH, such as loneliness and access to lethal means, as well as stressful life events such as sexual trauma. The SDoH concepts are based on standards established through the Gravity Project’s work on advancing SDoH and health equity data interoperability and customized by VA SMEs for the nuances of VA data, and the care needs specific to veterans.^8^ These standards support the consistent use of SDoH data across organizations, providers, and caregivers, and help to facilitate social risk data collection through intervention activities such as referrals, counseling, and care coordination.

The SDoH extracted in this project are being piloted as part of patient risk review dashboards to support mental health care delivery and will be used as features in the next iteration of predictive models used to target prevention interventions to patients with elevated suicide and overdose risk. One of these models is the Stratification Tool for Opioid Risk Mitigation (STORM), which estimates risk of opioid-related serious adverse events in the next year for VHA patients.^9^ STORM is used as a point of care decision support system that supports care for all VHA patients, facilitating risk benefit evaluation for patients considering opioid therapy and management of patients with behavioral health needs. Another model estimates the risk of suicide death in the next month for all VHA patients. It is used by the Recovery Engagement and Coordination for Health—Veterans Enabled Treatment (REACHVET) suicide prevention program to target case review and outreach programs to patients who are estimated within the top 0.1% of risk.^10^

### Importance of codesigning with clinical and technical experts

**Figure 1.**
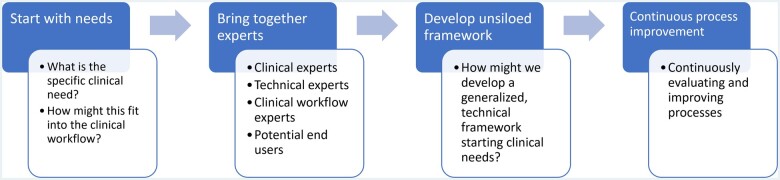
Steps of developed clinical text processing framework.

A core feature of our approach is codesigning with clinical experts and future users, as well as software and machine learning experts responsible for algorithm and pipeline development (Figure 1). A major barrier to operationalizing clinical text processing algorithms is that they often do not start with a core clinical need nor plan for clinical workflow integration.^4^ In this project, we ensure these critical factors were addressed from the outset. For CCTS, user needs center on daily identification of potentially missed cases of lung nodules within the software application to enable timely intervention by care coordinators. To facilitate judging accuracy, it was essential to highlight specific sections of the text identified by the text processing algorithm, enabling providers to easily locate and evaluate the results. These needs led the technical team to prioritize frequent model runs, establish data pipelines to necessary locations, and focus on named entity recognition models that allow highlighting of the relevant text, as opposed to text classification models. In the SDoH use case, there were multiple specific downstream clinical needs. The future integration of these extracted concepts into 2 risk algorithms already in production provided details on the data location and output format needed for the text processing models. Furthermore, clinical experts actively serving VHA patients provided input on which SDoH concepts may be most extractable and impactful to improve downstream risk assessment, as well as their validation for use within national clinical decision support tools.

### Importance of developing a system that works across projects

Another core feature of our approach is the design of frameworks that promote interoperability across projects, reducing the effort, cost, and variability between text processing implementations at VA. Traditionally, such projects have operated in isolation, with disparate teams creating custom implementations that often lack consideration for production requirements. By establishing a centralized infrastructure with daily access to cleaned, deidentified clinical notes, annotation tools, and production pipelines, we provide a common starting place for text processing initiatives. This centralization not only reduces computational and maintenance costs but also streamlines the development process. In the future, we expect this approach to improve discoverability and usability of validated text processing extractions across projects.

### HDAP text processing infrastructure

We developed an end-to-end system for developing and deploying text processing models at VA, with lung nodule identification and SDoH as initial use cases. Flexible custom code was developed to support both projects with minimal changes, creating a text processing solution that enables easy modification for future use cases. The main differences between the 2 projects are the data and initial entity extraction.

**Figure 2.**
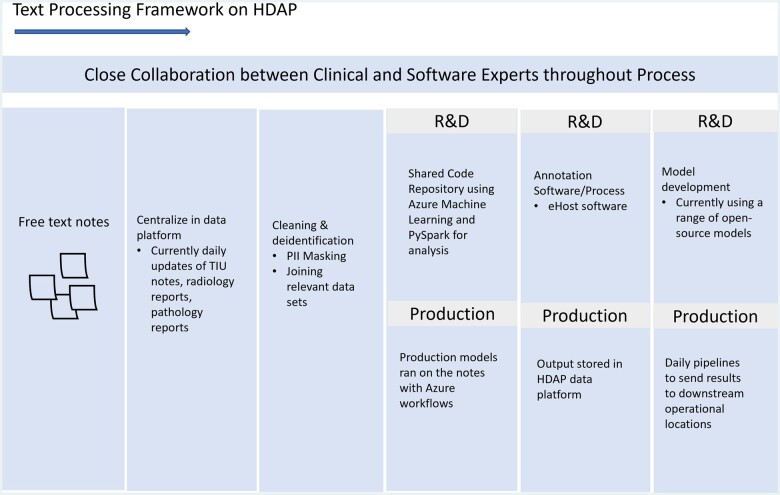
Use case development process.

This system is composed of 5 main steps (Table 1, Figure 2): data centralization, data pre-processing, data annotation, model training, and productionizing.

**Table 1.** Steps of developed clinical text processing framework.

Steps of text processing framework	
Data centralization	Daily update of free text notes into data platform
Data pre-processing	Joining relevant tables Special character removal PII masking
Data annotation	eHOST software Pre-highlighting of relevant terms
Model training	Pre-written scripts and workflows using open-source frameworks such as PyTorch,^11^ Nvidia Nemo,^12^ Azure Machine Learning,^13^ Hugging Face Transformers,^14^ Apache Spark,^15^ and Bidirectional Encoder Representations from Transformers (BERT) based models^16^
Productionizing	Daily model runs Pipelines connecting text processing output to operational applications Future: Promoting data products to be discoverable across projects

#### Step 1: Data centralization

We created a central repository in HDAP where free text notes are regularly updated and analyzed. We set up a workflow using Azure workflows to deposit a daily update of EMR notes (Radiology, Pathology, Text Integration Utility (TIU) (all other clinical progress notes)^17^). VA generates 1.5-2 million TIU notes per day, and the total size of all notes is 9 Terabytes compressed, so a big data platform like HDAP was crucial for the extraction and manipulation of these data.

#### Step 2: Data pre-processing

For both use cases, we extract about 150 million free text notes. Data were cleaned by removing special characters and masking personally identifiable information via a custom regex-based method. This pre-processed dataset is available for all projects, kickstarting the initial data gathering step and reducing the overall computation cost for projects using these datasets.

#### Step 3: Data annotation

After pre-processing, the projects differ for initial entity extraction. For SDoH, a clinical concept recognition tool, CLEVER, extracts free-text snippets containing SDoH terms from TIU notes to create the training and testing data.^18^^,^^19^ For CCTS, VA-based radiology subject matter experts identified terms for lung nodules and flags for reviewing follow-up cases. We also integrated the data annotation software eHOST for expert annotation to improve upon the initial labels for the models.^20^ We are currently working with subject matter experts to annotate terms for both projects.

#### Step 4: Model training

Training and inferencing were fine-tuned from a base Nvidia BioMegatron large language model using Azure Distributed GPU cluster computes.

#### Step 5: Productionizing

We have started productionizing models by establishing daily prediction runs using Azure Machine Learning and Azure Data Factory, ensuring models run as soon as the day’s data are available. We also set-up pipelines to transfer results to downstream systems. At VA, operational clinical applications run in a variety of environments—CDW is one of the most common locations and applications are increasingly being powered by HDAP. Since many applications run in CDW, including the mental health dashboards for the SDoH use case, we set-up a daily ETL from HDAP to CDW where text extractions are transferred and loaded into an on-prem CDW SQL database. For applications that run in HDAP, such as CCTS, extractions are loaded directly into a cloud SQL database. These 2 workflows provide the ability for text extractions to be integrated into a wide range of VA applications.

### Onboarding new use cases

We developed a system for onboarding new use cases that can leverage this infrastructure, detailed in Table 2. We have established a data science team on HDAP that assists with training, onboarding new use cases, and other support activities.

**Table 2.** Pathways for new use cases.

Onboarding new use cases		
Intake and onboarding	Users submit project intake request to be onboarded to HDAP.	
Data access	Currently available notes	Onboarded users have immediate access to the centralized and cleaned notes that we currently process (TIU, pathology, radiology) (from step 1).
	New types of notes	HDAP already has daily updates of all major free text notes in VA; however new types of notes can also be uploaded, if external, or pulled into our daily ETL processes, if they already exist elsewhere in our systems.
Data annotation	A supported data annotation environment (including eHOST) is available for use across projects with set-up and access supported by our internal HDAP data science team. Scripts and workflows are also available for document prepping, content loading and schema conversion, to send data to and from eHOST schemas. For the SDoH use case, a public website is used to support annotation and provides guidelines and concept definitions. This is the current link: http://ec2-18-206-230-88.compute-1.amazonaws.com/wordpress/?page_id=556	
Model training	We provide code and workflows for model training and fine-tuning from data annotation outputs, as well as a range of open-source models that have been brought into HDAP.	
Productionizing new use cases	Current downstream locations (HDAP and CDW)	When text processing algorithms are ready for productionization, they can be included in the current daily model running workflows and ETLs, with support from our HDAP data science team.
	New downstream operational locations	When necessary, additional ETLs to new locations can be established by the HDAP data science team.

## Discussion

Despite the plethora of text processing research in healthcare, few projects have bridged the gap from research to operations. To address this challenge, we implemented a text processing infrastructure within the VA, creating a framework that simplifies prototyping, deploying, and integrating text processing projects into healthcare operations. This project also sets up a framework for sharing resources (eg, data, code, production pipelines) among groups to reduce silos and lower the barrier to start future projects. Crucially, this project had software and machine learning experts working closely with clinical workflows and clinical experts, ensuring projects were grounded in clinical use cases and were developed with healthcare operations in mind from the beginning.

Overall, building text processing infrastructure that bridges use cases was a more efficient use of human and compute resources since many steps are shared between use cases. However, we identified a few needed customizations between use cases, particularly in the data term annotation process. To increase expert annotation speed, we pre-highlighted potential terms for annotators to accept, reject, or reject and highlight elsewhere, with the hypothesis that this pre-highlighting would save annotation time and allow more notes to be annotated. We found this approach worked well for the radiology use case, where the pre-highlighted terms were quite accurate and the term vocabulary was limited. However, for the SDoH use case, we found that auto-highlighting note text resulted in the annotators reviewing the notes too quickly, leading to low-quality annotations. This was likely due to the relative complexity of annotating SDoH data from unstructured EHR data versus more established biomedical concepts, such as nodule size, character, or change in dimension. This demonstrates the importance of testing the annotation steps for each unique use case first to identify the most accurate approach.

The next steps are to continue improving the text processing algorithms and infrastructure, including integrating use cases directly into healthcare operations, and establishing a framework for model monitoring and evaluation of clinical benefit while also enhancing the framework’s usability and scalability. We are expanding the use cases on our platform, having onboarded 3 more use cases over the past few months. Furthermore, we are developing governance strategies for text extractions from EHR data to make them more useable and discoverable across VA.

## Data Availability

There are not specific data to release related to this project.

## References

[1] Patra BG , Sharma MM, Vekaria V, et al Extracting social determinants of health from electronic health records using natural language processing: a systematic review. J Am Med Inform Assoc. 2021;28(12):2716-2727. 34613399 10.1093/jamia/ocab170 PMC8633615

[2] Sy LW , Osorio C, Huang J. An empirical evaluation of deep learning for ICD-9 code assignment using MIMIC-III clinical notes. Comput Methods Programs Biomed. 2019;177:141-153. 31319942 10.1016/j.cmpb.2019.05.024

[3] Li I , Pan J, Goldwasser J, et al Neural natural language processing for unstructured data in electronic health records: a review. Comput Sci Rev. 2022;46:100511.

[4] Lederman A , Lederman R, Verspoor K. Tasks as needs: reframing the paradigm of clinical natural language processing research for real-world decision support. J Am Med Inform Assoc. 2022;29(10):1810-1817. 35848784 10.1093/jamia/ocac121 PMC9471702

[5] Tamang S , Humbert-Droz M, Gianfrancesco M, et al Considerations for developing clinical natural language processing systems for population health management and measurement. JMIR Med Inform. 2023;11(1):e37805. 36595345 10.2196/37805 PMC9846439

[6] Wen A , Fu S, Moon S, et al Desiderata for delivering NLP to accelerate healthcare AI advancement and a Mayo Clinic NLP-as-a-service implementation. NPJ Digit Med. 2019;2(1):130. 31872069 10.1038/s41746-019-0208-8 PMC6917754

[7] Zhang Y , Mezzacappa C, Shen L, et al Cancer tracking system improves timeliness of live cancer care at a Veterans Hospital: a comparison of cohorts before and after implementation of an automated care coordination tool. PLoS Digit Health. 2022;1(8):e0000080. 36812575 10.1371/journal.pdig.0000080 PMC9931271

[8] The Gravity Project. Accessed October 28, 2023. https://thegravityproject.net

[9] Oliva EM , Bowe T, Tavakoli S, et al Development and applications of the Veterans Health Administration’s Stratification Tool for Opioid Risk Mitigation (STORM) to improve opioid safety and prevent overdose and suicide. Psychol Serv. 2017;14(1):35. 10.1037/ser0000099 28134555

[10] McCarthy JF , Cooper SA, Dent KR, et al Evaluation of the recovery engagement and coordination for health-veterans enhanced treatment suicide risk modeling clinical program in the Veterans Health Administration. JAMA Netw Open. 2021;4(10):e2129900. 34661661 10.1001/jamanetworkopen.2021.29900 PMC8524305

[11] Paszke A , Gross S, Massa F, et al Pytorch: an imperative style, high-performance deep learning library. Adv Neural Inf Process Syst. 2019;32:1-12.

[12] Kuchaiev O , Li J, Nguyen H, et al 2019. Nemo: a toolkit for building ai applications using neural modules, arXiv, arXiv:1909.09577, preprint: not peer reviewed.

[13] Azure Machine Learning. Accessed June 23, 2023. https://azure.microsoft.com/en-us/products/machine-learning

[14] Wolf T , Lysandre D, Sanh V, et al 2019. Huggingface’s transformers: state-of-the-art natural language processing, arXiv, arXiv:1910.03771, preprint: not peer reviewed.

[15] Zaharia M , Xin RS, Wendell P, et al Apache spark: a unified engine for big data processing. Commun ACM. 2016;59(11):56-65.

[16] Devlin J , Chang MW, Lee K, et al BERT: pre-training of deep bidirectional transformers for language understanding. In: *Proceedings of the 2019 Conference of the North American Chapter of the Association for Computational Linguistics: Human Language Technologies, NAACL-HLT*, Minneapolis, MN. Association for Computational Linguistics; 2019:4171-4186.

[17] Department of Veterans Affairs Text Integration Utilities (TIU) Technical Manual—May 2023. Accessed July 15, 2023. https://www.va.gov/vdl/documents/Clinical/CPRS-Text_Integration_Utility_(TIU)/tiutm.pdf

[18] Tamang SR , Hernandez-Boussard T, Ross E, et al Enhanced quality measurement event detection: an application to physician reporting. EGEMS (Wash DC). 2017;5(1):5. 10.13063/2327-9214.1270 PMC5983066 29881731

[19] Ling AY , Kurian AW, Caswell-Jin JL, et al Using natural language processing to construct a metastatic breast cancer cohort from linked cancer registry and electronic medical records data. JAMIA Open. 2019;2(4):528-537. 32025650 10.1093/jamiaopen/ooz040 PMC6994019

[20] extensible Human Oracle Suite of Tools (eHOST). Accessed July 15, 2023. https://www.github.com/chrisleng/ehost

